# HybTrack: A hybrid single particle tracking software using manual and automatic detection of dim signals

**DOI:** 10.1038/s41598-017-18569-3

**Published:** 2018-01-09

**Authors:** Byung Hun Lee, Hye Yoon Park

**Affiliations:** 1 Department of Physics and Astronomy, Seoul National University, Seoul, 08826 Korea; 2 Institute of Applied Physics, Seoul National University, Seoul, 08826 Korea

## Abstract

Single particle tracking is a compelling technique for investigating the dynamics of nanoparticles and biological molecules in a broad range of research fields. In particular, recent advances in fluorescence microscopy have made single molecule tracking a prevalent method for studying biomolecules with a high spatial and temporal precision. Particle tracking algorithms have matured over the past three decades into more easily accessible platforms. However, there is an inherent difficulty in tracing particles that have a low signal-to-noise ratio and/or heterogeneous subpopulations. Here, we present a new MATLAB based tracking program which combines the benefits of manual and automatic tracking methods. The program prompts the user to manually locate a particle when an ambiguous situation occurs during automatic tracking. We demonstrate the utility of this program by tracking the movement of β-actin mRNA in the dendrites of cultured hippocampal neurons. We show that the diffusion coefficient of β-actin mRNA decreases upon neuronal stimulation by bicuculline treatment. This tracking method enables an efficient dissection of the dynamic regulation of biological molecules in highly complex intracellular environments.

## Introduction

Single molecule techniques using bright fluorophores have been indispensable for studying complex biological systems over the last 20 years. With the tremendous advances in fluorescence microscopy technology, one can image single molecules with a spatial resolution of a few tens of nanometers in living cells^1^. Single particle tracking (SPT) is one of the crucial computational tools for quantitative analysis of the movement of various objects in time-lapse images^2,3^. Hence, there have been continuous efforts to develop more accurate, reliable and user-friendly software for tracking single molecules, organelles, or cells for biological studies^4–6^.

Because manual tracking is labor intensive and often infeasible for large datasets, most of the particle tracking software programs use automated algorithms for particle detection and linking. A number of SPT software packages have been developed using particle detection approaches such as simple thresholding, centroid estimation, and Gaussian model fitting, in combination with various particle linking methods such as simple nearest-neighbor, multiple hypothesis tracking, Kalman filtering, and combinatorial optimization^4,6^. Those automatic software tools often give different tracking results on the same dataset depending on the algorithms and parameter settings, making it challenging to choose the correct tracking result. An objective comparison of 14 different SPT software tools found that there is no single universally best method for particle tracking^4^. Thus, users need to use their best knowledge to determine which software tool is appropriate for their specific biological problems and should be especially careful when the signal-to-noise ratio (SNR) of their data is lower than 4^4,7,8^.

Because single molecule imaging has inherently low signal levels and the inevitable problem of photo-bleaching, one often has to deal with dim images with a low SNR. For low-SNR data, more complicated automatic tracking methods such as multi-frame optimization and well-tuned motion models perform better than simpler methods using two-frame linking and nearest-neighbor search^4^. However, when software becomes more complex, the user needs to optimize more parameters for their specific condition. Although the tracking process itself is performed automatically, researchers have to examine the tracking data and re-adjust the parameters until they find a tracking result that matches their perceived trajectories of the particles. Such an iterative process can result in an even longer analysis time than the manual tracking process. Furthermore, particles in the image may reside in different microenvironments inside a cell, which requires different tracking parameter settings for each particle.

Here, we developed HybTrack, a novel software tool that enables the tracking of dim particles using a combination of manual and automatic detection. Rather than leaving the whole process to an automatic algorithm, HybTrack provides the user an opportunity to participate in particle tracking. To our knowledge, this is the first particle tracking software that allows switching between manual and automatic detection. We demonstrate that a little intervention using manual selection can dramatically improve the performance of particle tracking. Analysis of β-actin mRNA transport in neurons highlights the advantages of HybTrack to track heterogeneous populations of particles with a low SNR.

## Results

### Overview of HybTrack

**Figure 1:**
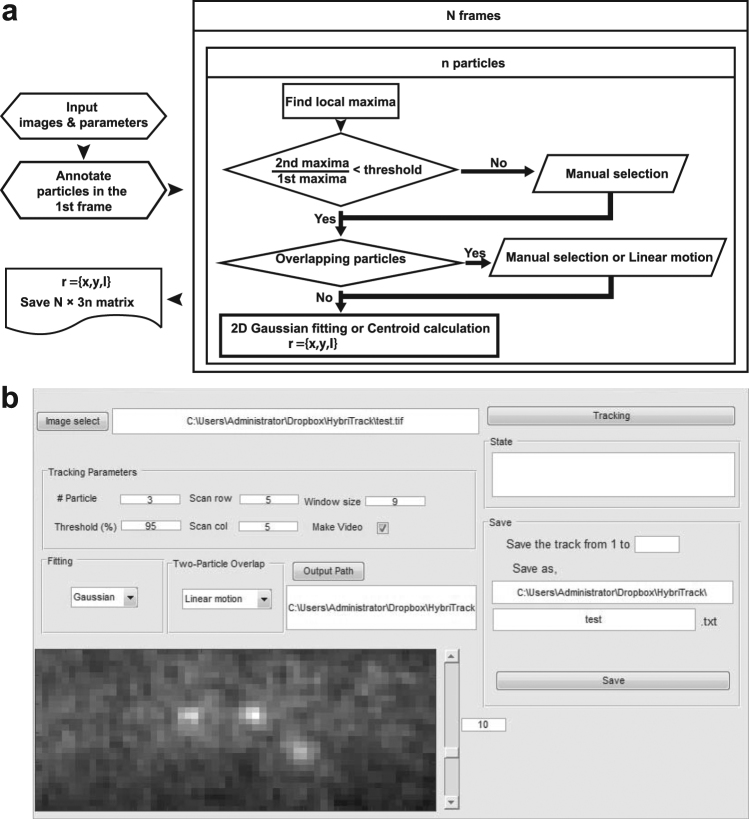
Flow chart and graphical user interface (GUI) of HybTrack. (**a**) Tracking is started by selecting the image and setting input parameters. The user needs to annotate particles to track in the first image frame. Based on the initial positions, the tracking algorithm proceeds to search for local maxima and calculates the sub-pixel coordinates. If the local maxima is not bright enough, a pop-up window appears for manual detection of the particle. If there are overlapping particles within the scan region, two options are provided, Manual selection or Linear motion. This process is repeated for all annotated particles and image frames. (**b**) GUI interface of HybTrack. After setting the parameters, tracking process is started, and the result is saved as a text file.

Most of the automatic tracking programs generally use a two-step process: (i) detection of particles in all image frames, and then (ii) linking of the particles in consecutive images. In contrast, HybTrack performs particle detection and tracking simultaneously frame by frame (Fig. 1). In each image frame, tracking of a particle is performed with the following procedure: (i) selecting a particle scan region, (ii) detecting local maxima in the scan region and sub-pixel localization of the particle, and (iii) saving the particle position and updating the scan region for the particle in the next frame (see Supplementary Note and Supplementary Fig. S1).

At the beginning of the tracking procedure, the user provides the number of particles to track, and the initial positions of those particles are selected by clicking on the bright spots in the first image frame (Supplementary Fig. S2). Based on this information, the program defines a particle scan area which has a height and width of the pre-defined parameter, ***Scan row*** and ***Scan col***. Because HybTrack typically works with a small scan area (***Scan row*** ≈ ***Scan col*** ≈ 5–50 pixels), image filtering is generally not required to detect local maxima within the search region. Local maxima are found by calculating the mean intensity of all rectangles with the size parameter ***Window size*** within the search area. After a local maximum is found, the sub-pixel position of the particle is calculated by the centroid or by fitting the image with a two-dimensional (2D) Gaussian function. Finally, the particle position is saved and used to define a new scan area for the next frame.

If the image data have a sufficiently high SNR and the particles exhibit small movements, HybTrack completes automatic tracking without any interruption. However, when there is an ambiguity in the automatic particle tracking, a pop-up window appears for manual tracking (Supplementary Fig. S3). There are two representative cases where manual tracking is required. First, the particle image could be too dim or noisy to detect local maxima automatically. Even though computer algorithms fail to detect such a dim signal, human vision can sometimes distinguish a particle out of a noisy background. Therefore, we implemented the HybTrack software to provide the user an opportunity to examine the image. If no particle was detected automatically in the scan area, HybTrack offers three options: ***Stop***, ***Manual detection***, and ***Gap***. By choosing ***Stop***, the user can terminate the trajectory of the corresponding particle. If ***Manual detection*** is selected, a new window pops up so that the user can select the position of the particle manually in the image. The ***Gap*** option leaves the position of the particle as a NaN (Not-a-Number) value. The second case that requires manual tracking is when two particles are found within a scan region. In this case, HybTrack provides two options under ***Two-particle overlap***. One option is to select each particle’s position manually in the image. The other one is the ***Linear motion*** option which predicts the particle’s position based on the previous velocity of the particle. Then the predicted spot is used as an approximate position for sub-pixel localization of the particle. The ***Linear motion*** option is useful when a particle exhibits directed motion with a constant velocity during an overlapping event (Supplementary Fig. S4).

### Tracking single mRNA in live neurons

To demonstrate the utility of the HybTrack software, we performed single particle tracking of β-actin mRNA in live hippocampal neurons. By imaging neurons cultured from MCP × MBS mice which express GFP-labeled β-actin mRNA^9^, we observed the real-time dynamics of single β-actin mRNA molecules. In the MCP × MBS mouse, 24 repeats of the MS2 binding site (MBS) stem-loop are inserted in the 3′ untranslated region (3′UTR) of the endogenous β-actin gene^10^. The mouse also expresses the MS2 capsid protein (MCP) fused with GFP (MCP-GFP), a dimer of which binds to an MBS stem-loop with high specificity and affinity. Thus, each β-actin mRNA is labeled with up to 48 GFPs and becomes bright enough for single mRNA imaging albeit the background of free MCP-GFPs in the cytoplasm.

**Figure 2:**
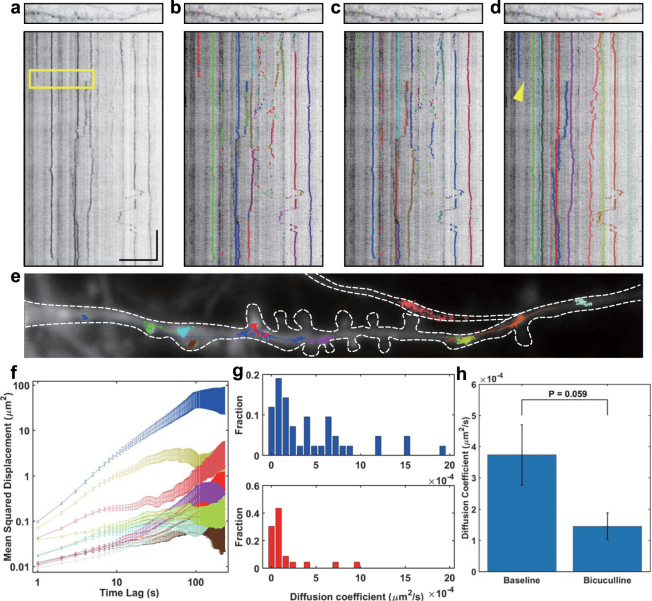
Application of HybTrack to track mRNAs in neurons. (**a**) Image of mRNAs in a dendrite and kymograph (x-t) generated from the time-lapse image. Scale bar, 10 µm (horizontal) and 1 min (vertical). (**b**–**d**) Tracking results from u-Track (**b**), TrackNTrace (**c**) and HybTrack (**d**). Particle trajectories obtained from each program are overlaid on the image (upper panels) and the kymograph (lower panels). Each trajectory is shown in a different color. (**e**) mRNA trajectories detected by HybTrack are plotted on the time-averaged image. White dashed lines outline the dendrite. (**f**) The mean squared displacement of 10 mRNAs detected by HybTrack. The error bars are calculated as described in the Methods section. (**g**) Histograms of diffusion coefficient of diffusive mRNAs in the baseline (upper panel) and after bicuculline treatment (lower panel). (**h**) Effect of bicuculline treatment on the mean diffusion coefficient of diffusive mRNAs in the dendrites. Error bars represent SEM (n = 5 dendrites; *P* = 0.059, pairwise t-test).

Figure 2a shows an image of a dendritic segment and a kymograph generated from a time-lapse movie of a dendrite (Supplementary Movie S1). Projected on the y plane of x-y-time voxels, the kymograph shows single mRNA paths along the dendrite. Most of the β-actin mRNAs in the neurons are stationary, but some mRNAs show diffusive or directed motion^11^ and occasionally change their motion types^12^. Moreover, a moving mRNA sometimes gets out of the imaging plane or overlaps with another mRNA. Although automatic algorithms have been developed to address these cases, manual tracking can be a more direct and easier way to handle them.

To assess the performance of HybTrack, we compared the tracking results with those from two state-of-the-art automatic tracking programs, u-Track^13^ and TrackNTrace^14^. u-Track is one of the most widely used single particle tracking (SPT) program, and TrackNTrace is one of the latest tracking programs which offers an extendable open-source framework for various applications. To compare the tracking results, we plotted the traces obtained by each program on the kymograph (Fig. 2b–d). It is evident that even highly sophisticated automatic tracking programs suffer from incomplete linking of particle trajectories that have a low SNR. Although human eyes can trace about 10 particles in the image shown in Fig. 2a, u-Track and TrackNTrace recognized them as 23 and 25 different particles (with a track length >20 frames), respectively. However, HybTrack was able to construct the full trajectories of 10 particles that had a low SNR ranging from 1.15 to 3.65 (Fig. 2d). Supplementary Fig. S5 also shows an example of tracking very dim particles.

Another advantage of HybTrack can be found when tracking particles showing directed motion with a relatively high speed. For example, in Fig. 2a, the mRNA on the far-left travels to the middle of the image (yellow box) at a speed of 1.3 μm/s. During the directed motion, the mRNA traveled much faster than the other mRNAs and left only sparse spots. By using the manual tracking option in HybTrack, those sparse spots can be linked with just a few clicks (Fig. 2d, yellow arrow). Another example of linking directed motion is shown in Supplementary Fig. S6. In this 460-frame-long time-lapse image, HybTrack successfully tracked three mRNA particles with only 6 clicks of manual detection.

In Fig. 2e, particle trajectories obtained by HybTrack are overlaid on the dendrite image shown in Fig. 2a. Most of the β-actin mRNAs were localized near the dendritic spine necks or inside the spines suggesting that the local translation of β-actin has a role in stabilizing the dendritic spines^15^. The time-averaged mean squared displacement (TA-MSD) of each mRNA is plotted in log-log scale in Fig. 2f. The TA-MSD of the mRNA that showed directed motion (blue line) has an exponent larger than 1, indicating super-diffusive motion. However, most of the mRNAs exhibited sub-diffusive motion with exponents less than 1. The heterogeneous nature of the mRNA movement is also demonstrated by the wide distribution of the diffusion coefficients in Fig. 2g.

Finally, we investigated the activity-dependent dynamics of β-actin mRNA by tracking dendritic mRNAs using HybTrack. To investigate the changes in the movement of mRNA upon stimulation, we treated hippocampal neurons from MCP × MBS mice with bicuculline which is a GABA receptor blocker. We performed tracking of mRNAs in the same dendrite before and after stimulation and calculated the diffusion coefficients of diffusive mRNAs. After the treatment with bicuculline, there was a significant decrease in the diffusion coefficients (Fig. 2g; n = 44 mRNAs in the baseline, n = 23 mRNAs after bicuculline treatment; *P*_*KS*_ = 0.0013, Kolmogorov-Smirnov test). The mean diffusion coefficient of the mRNAs in the dendrites also decreased upon stimulation (Fig. 2h; n = 5 dendrites; *P* = 0.059, pairwise t-test). This result is consistent with a previous report which showed a decrease in the diffusion coefficient of β-actin mRNA upon neuronal stimulation by KCl depolarization^9^. These observations suggest that β-actin mRNAs may be anchored by so-called synaptic tags^16^, which are expected to be identified in the future.

## Discussion

We have developed HybTrack as a new practical tool for the analysis of single particle imaging data. Fully automated tracking often fails to capture the entire trajectory of a particle visible to the researcher. Manual tracking software such as MTrackJ^5^ offers flexible track editing functionalities for trajectory inspection and curation. However, it would be extremely tedious to manually follow each particle through hundreds to thousands of image frames. HybTrack facilitates tracking of single particles that have a low SNR by combining the advantages of both automatic and manual tracking methods. The combination of these two methods enables our algorithm to give results closest to the human vision with high efficiency.

While automatic particle tracking programs generate tracks by constructing paths for all detected particles, our algorithm generates tracks starting from the initial particle positions selected by the user in the first image frame. Starting with manual selection substantially reduces the interference from noise. There have been a couple of semi-automatic particle tracking software^17,18^ that require particle annotation in the first image frame and perform automated tracking afterwards. However, HybTrack is the first kind of single particle tracking program, to our knowledge, that enables switching between manual and automatic detection during frame-by-frame tracking of individual particles. For this reason, HybTrack is very useful for efficient tracking of a highly heterogenous population of particles that alternate between different motion types. Such trajectories obtained from HybTrack can be subsequently processed by an objective analysis method such as MSD-Bayes approach^12,19^ to automatically classify particle motions.

A limitation of HybTrack is that it may not be suitable for high-throughput analysis of numerous particles in a single data set. While other automatic tracking algorithms would be advantageous to analyze the overall behavior of many particles, HybTrack is more useful for the precise analysis of individual particle trajectories. For example, our algorithm can be readily applied to analyze the movement of single particles with respect to subcellular components such as nuclear pores^20^, focal adhesions^21,22^, P-bodies^23,24^, cytoskeletons^25,26^, and so forth. We expect that this new software will be a valuable addition to the SPT analysis tools to solve many outstanding problems in single-cell single-molecule biology.

## Methods

### Software

The tracking software used in this work is available at http://github.com/bhlee1117/HybTrack/. The software details are described in the Supplementary Note.

### Primary mouse neuron cultures

All animal experiments were conducted in accordance with methods approved by the Institutional Animal Care and Use Committee (IACUC) at Seoul National University. Primary hippocampal neurons were cultured from 1-day-old pups of the MCP × MBS mice^9^ using a method described previously^27^. Briefly, hippocampi were dissected out from the brains of 3–4 pups and dissociated by trypsin. Glass-bottom dishes were coated with poly-D-lysine, on which ~10^5^ dissociated neurons were seeded. The neuron cultures were grown for 8–14 days *in vitro* in Neurobasal-A medium (Gibco) supplemented with B-26 (Gibco), Glutamax (Gibco) and Primocin (Invivogen) at 37 °C and 5% CO_2_.

### Imaging single mRNA in live neurons

Live neuron imaging experiments were performed as described previously^27^. Prior to the imaging, culture medium was removed from the neuron culture and replaced with HEPES-buffered saline (HBS) containing 119 mM NaCl, 5 mM KCl, 2 mM CaCl_2_, 2 mM MgCl_2_, 30 mM D-glucose, and 20 mM HEPES at pH 7.4. Wide-field fluorescence images were taken with U Apochromat 150 × 1.45 NA TIRF objective (Olympus) on an Olympus IX73 inverted microscope equipped with an iXon Ultra 897 electron-multiplying charge-coupled device (EMCCD) camera (Andor), an MS-2000 XYZ automated stage (ASI), and Chamlide TC top-stage incubator system (Live Cell Instrument). A 488-nm diode laser (Cobolt) was used to excite the GFP, and the fluorescence emission was filtered with a 525/50 band-pass filter (Chroma). Time-lapse images were taken at 10 frames per second (fps) with the Micro-Manager software.

### Image analysis

2D Gaussian fitting was performed with a weighted overdetermined regression method^28^. Background-subtracted fluorescence images were fit with

1 $${\rm{I}}({\rm{x}},{\rm{y}})=A\,\exp (-\frac{{(x-{x}_{c})}^{2}}{2{w}_{x}^{2}}-\frac{{(y-{y}_{c})}^{2}}{2{w}_{y}^{2}})$$

where *A* is the peak amplitude; *x*_*c*_ and *y*_*c*_ are the center position of the Gaussian function, and *w*_*x*_ and *w*_*y*_ are the standard deviations in the x and y coordinates. In HybTrack, the output intensity value is calculated by the integration of the 2D Gaussian function $2\pi A{w}_{x}{w}_{y}$.

In the centroid method, the particle location was obtained by

2 $${{\rm{x}}}_{{\rm{c}}}=\frac{{\sum }_{i,j}{I}_{ij}\cdot {x}_{i}}{{\sum }_{i,j}{I}_{ij}},\,{y}_{c}=\frac{{\sum }_{i,j}{I}_{ij}\cdot {y}_{j}}{{\sum }_{i,j}{I}_{ij}}$$

where *x*_*i*_ and *y*_*j*_ are the position of the corresponding pixel in the particle image cropped by the ***window size***. The output intensity value was calculated by

3 $$({\rm{sum}}\,{\rm{of}}\,{\rm{intensity}}\,{\rm{in}}\,{\rm{the}}\,{\rm{window}})-({\rm{background}}\,{\rm{intensity}})\times ({\rm{window}}\,{\rm{size}})$$

The SNR of a particle image was calculated by

4 $${\rm{SNR}}=\frac{{{\rm{\mu }}}_{{\rm{sig}}}-{{\rm{\mu }}}_{{\rm{background}}}}{{{\rm{\sigma }}}_{{\rm{background}}}}$$

where ${{\rm{\mu }}}_{{\rm{sig}}}$ and ${{\rm{\mu }}}_{{\rm{background}}}$ are the mean intensity value of the signal and background region respectively, and ${{\rm{\sigma }}}_{{\rm{background}}}$ is the standard deviation of the background region.

### Calculation of mean squared displacement (MSD)

The MSD denoted as ${\rho }_{n}$ of N-frame-long particle trajectory, ${\rm{r}}(i{\rm{\Delta }}t)=[{x}_{i},{y}_{i}],(i=1,2,\,\ldots ,N)$, was calculated by

5 $${{\rm{\rho }}}_{{\rm{n}}}={\rm{\rho }}(n{\rm{\Delta }}t)={\sum }_{i=1}^{N-n}({({x}_{i+n}-{x}_{i})}^{2}+{({y}_{i+n}-{y}_{i})}^{2})/(N-n)$$

In Fig. 2f, the relative error of MSD was calculated following Qian *et al*.^29^:

6 $${(\frac{{\rm{\Delta }}{\rho }_{n}{\rm{\Delta }}{\rho }_{n}}{\langle {\rho }_{{\rm{n}}}\rangle })}^{\frac{1}{2}}=\{\begin{array}{cc}{\{\frac{4{n}^{2}N-5{n}^{3}+2N-n}{6n{(N-n)}^{2}}\}}^{\frac{1}{2}} & n\le \frac{N}{2}\\ {\{1+\frac{{N}^{3}-5{n}^{3}-7n{N}^{2}+11{n}^{2}N+5n-N}{6{n}^{2}(N-n)}\}}^{\frac{1}{2}} & n > \frac{N}{2}\end{array}$$

where ${{\rm{\Delta }}{\rm{\rho }}}_{{\rm{n}}}$ denotes ${{\rm{\rho }}}_{n}-\langle {{\rm{\rho }}}_{{\rm{n}}}\rangle$, and N is the total frame number.

The diffusion coefficient of each mRNA was calculated by linear fitting of the MSD data.

### Data availability

Image data of β-actin mRNA in live neurons analyzed in the current study are available from the corresponding author upon reasonable request.

## Electronic supplementary material

Supplementary Information

Supplementary Movie S1

Supplementary Movie S2

Supplementary Movie S3

Supplementary Movie S4

## References

[1] Vera M, Biswas J, Senecal A, Singer RH, Park HY (2016). Single-Cell and Single-Molecule Analysis of Gene Expression Regulation. Annu Rev Genet.

[2] Manzo C, Garcia-Parajo MF (2015). A review of progress in single particle tracking: from methods to biophysical insights. Rep Prog Phys.

[3] Shen H (2017). Single Particle Tracking: From Theory to Biophysical Applications. Chem Rev.

[4] Chenouard N (2014). Objective comparison of particle tracking methods. Nat Methods.

[5] Meijering E, Dzyubachyk O, Smal I (2012). Methods for Cell and Particle Tracking. Method Enzymol.

[6] Park HY, Buxbaum AR, Singer RH (2010). Single mRNA tracking in live cells. Methods in enzymology.

[7] Cheezum MK, Walker WF, Guilford WH (2001). Quantitative comparison of algorithms for tracking single fluorescent particles. Biophys J.

[8] Smal I, Loog M, Niessen W, Meijering E (2010). Quantitative Comparison of Spot Detection Methods in Fluorescence Microscopy. Ieee T Med Imaging.

[9] Park HY (2014). Visualization of dynamics of single endogenous mRNA labeled in live mouse. Science.

[10] Lionnet T (2011). A transgenic mouse for *in vivo* detection of endogenous labeled mRNA. Nat Methods.

[11] Buxbaum AR, Yoon YJ, Singer RH, Park HY (2015). Single-molecule insights into mRNA dynamics in neurons. Trends Cell Biol.

[12] Monnier N (2015). Inferring transient particle transport dynamics in live cells. Nat Methods.

[13] Jaqaman K (2008). Robust single-particle tracking in live-cell time-lapse sequences. Nat Methods.

[14] Stein SC, Thiart J (2016). TrackNTrace: A simple and extendable open-source framework for developing single-molecule localization and tracking algorithms. Sci Rep.

[15] Yoon YJ (2016). Glutamate-induced RNA localization and translation in neurons. Proc Natl Acad Sci USA.

[16] Doyle M, Kiebler MA (2011). Mechanisms of dendritic mRNA transport and its role in synaptic tagging. EMBO J.

[17] Smith MB (2011). Interactive, computer-assisted tracking of speckle trajectories in fluorescence microscopy: application to actin polymerization and membrane fusion. Biophys J.

[18] Tinevez JY (2017). TrackMate: An open and extensible platform for single-particle tracking. Methods.

[19] Monnier N (2012). Bayesian approach to MSD-based analysis of particle motion in live cells. Biophys J.

[20] Grunwald D, Singer RH (2010). *In vivo* imaging of labelled endogenous beta-actin mRNA during nucleocytoplasmic transport. Nature.

[21] Katz ZB (2012). beta-Actin mRNA compartmentalization enhances focal adhesion stability and directs cell migration. Genes Dev.

[22] Katz, Z. B. *et al*. Mapping translation ‘hot-spots’ in live cells by tracking single molecules of mRNA and ribosomes. *Elife* **5**, 10415, 10.7554/eLife.10415 (2016). 10.7554/eLife.10415 PMC4764586 26760529

[23] Aizer A (2014). Quantifying mRNA targeting to P-bodies in living human cells reveals their dual role in mRNA decay and storage. J Cell Sci.

[24] Halstead JM (2015). Translation. An RNA biosensor for imaging the first round of translation from single cells to living animals. Science.

[25] Tanenbaum ME, Gilbert LA, Qi LS, Weissman JS, Vale RD (2014). A protein-tagging system for signal amplification in gene expression and fluorescence imaging. Cell.

[26] Pichon X (2016). Visualization of single endogenous polysomes reveals the dynamics of translation in live human cells. J Cell Biol.

[27] Moon HC, Park HY (2016). Imaging Single mRNA Dynamics in Live Neurons and Brains. Methods in enzymology.

[28] Anthony SM, Granick S (2009). Image analysis with rapid and accurate two-dimensional Gaussian fitting. Langmuir.

[29] Qian H, Sheetz MP, Elson EL (1991). Single particle tracking. Analysis of diffusion and flow in two-dimensional systems. Biophys J.

