# Supplementary material for: HybTrack: A hybrid single particle tracking software using manual and automatic detection of dim signals
Source: Sci Rep. 2018 Jan 9;8:212. doi: 10.1038/s41598-017-18569-3 (PMC5760724; doi:10.1038/s41598-017-18569-3)
Supplement: Supplementary file 1 — Supplementary Information [file 41598_2017_18569_MOESM1_ESM.pdf]

Supplementary Information for

HybTrack: A hybrid single particle tracking software using manual and automatic detection of dim signals

Byung Hun Lee[1] and Hye Yoon Park[1,2]*

Affiliations:

[1] Department of Physics and Astronomy, Seoul National University, Seoul, 08826, Korea

[2] Institute of Applied Physics, Seoul National University, Seoul, 08826, Korea

*Correspondence: hyeyoon.park@snu.ac.kr

This file includes:

Supplementary Note

Supplementary Figures S1 to S6

Captions for Supplementary Movies S1 to S4

Reference

Other supplementary material for this manuscript includes the following:

Supplementary Movies S1 to S4

Supplementary Note

1. Instructions for using the GUI version of HybTrack

1.1 Load a particle image

Download "HybTrack_GUI_version.zip" from http://github.com/bhlee1117/HybTrack/. Start MATLAB R2017a or newer version and go to the directory that contains the extracted files. From the MATLAB command prompt, launch the HybTrack_GUI (Supplementary Fig. 1) by typing:

```
>> HybTrack_GUI
```

Load a time-lapse image file by clicking the ***Image select*** button. For instance, select "test.tif" file included in the HybTrack_GUI software package. After you select the image, the image file name appears in the panel on the right side of the button. At the bottom of the "HybTrack_GUI" window, you can view the loaded time-lapse image by clicking the upper arrow of the slider bar.

1.2 Choose options and settings

# Particle: This parameter determines how many particles will be tracked. For instance, type "3" to track three particles in "test.tif".

Scan row and ***Scan col***: These parameters determine the height and width of the scanning region. When a particle position is detected in a frame, HybTrack will search local maxima in the next frame in the scanning region around the previous position. Therefore, these parameters need to be determined by considering the overall range of the particle movement. The default values are 5 pixels for the particles in "test.tif".

Window size: This parameter is defined by the value N x N, where N is an odd integer smaller than

Scan row and ***Scan col*** (e.g., ***Window size*** = 1, 9, 25 …). When searching for the local maxima in the scanning region, HybTrack compares the mean intensity values of all windows, each of which has the ***Window size*** and centers at each pixel in the scanning region. If ***Window size*** is too big, the localization error becomes large. If ***Window size*** is too small, tracking will be interfered by noise. The default value is 9 pixels for the particles in "test.tif".

Threshold (%): This parameter determines how often the manual tracking option will be available. After the mean intensity of the windows are calculated, the two largest values are compared. If the

$\frac{2^{nd}\text{ largest mean value}}{\text{largest mean value}}$ is smaller than ***Threshold*** (%), HybTrack determines the local maximum at the pixel with the largest mean value. The lower the ***Threshold***, the more frequently the pop-up window for manual tracking will appear. The default value for "test.tif" is 95%.

Make Video: Check this box if you want to watch the tracking procedures and save the tracking video.

Fitting: HybTrack provides two options for sub-pixel localization. One is two-dimensional (2D) Gaussian fitting using a weighted overdetermined regression method (Anthony and Granick 2009). If the images are too noisy to fit with the 2D Gaussian function, the user can choose the other option to calculate the centroid.

Two-Particle Overlap: When two particles are detected in the same scan region, HybTrack provides two options. One is the manual tracking option, which prompts the user to select the two positions of the particles in the image (Supplementary Fig. 3). The other one is the "Linear motion" option, which predicts the particle position based on the history of the trajectory. The particle is assumed to move with a constant velocity calculated from the 10 previous frames. Then the predicted position is used as an approximate location instead of local maxima for sub-pixel localization of the particle. The result of using the "Linear motion" option is shown in Supplementary Fig. 4. The directed motion of the green particle was successfully traced automatically despite the overlapping event with the blue particle.

Output Path: Choose the directory in which the result files (tracking files and videos) will be saved.

1.3 Detection and tracking

After setting the parameters, click the ***Tracking*** button. Then, you will see a new window which prompts you to select the particles to track (Supplementary Fig. 2). For example, three particle positions (red circles in Supplementary Fig. 2) can be selected in "test.tif". After clicking the number of positions same as ***# Particle***, the tracking process will start.

If ***Make Video*** is checked, the tracking procedure will be shown. The frame number being processed is shown in the ***State*** panel below the ***Tracking*** button. When there are zero or two particles in the scan region, a pop-up window will show up (Supplementary Fig. 3). In the pop-up

window, there are three options to choose. ***Stop*** button ends the tracking of the particle. ***Manual detection*** button enables the user to select the position of the particle manually. ***Gap*** button enters the NaN value for the corresponding tracking position. This iterating procedure continues to the last frame of the image.

1.4 Save results

When tracking is complete, HybTrack shows the particle trajectories overlaid on the image. If the tracking result looks good, the user can save the result in the folder designated by the ***Output Path***. Because of photo-bleaching, the later part of the image could be too dim for proper tracking. To cut those data, the user can edit the last frame number to save. The name of result file can be also edited in the input panel. After editing is complete, the user can save the file by clicking the ***Save*** button.

The saved text file has (3 × # ***particles***) columns. The first three columns contain the x position, y position, and the intensity of the first particle. And the next three columns are for the second particle, and so forth.

2. Instructions for using the script version of HybTrack

Download "HybTrack_script_version.zip" from http://github.com/bhlee1117/HybTrack/. Start MATLAB R2017a or newer version and go to the directory that contains the extracted files. Open "Hybtracking_script.m" in the MATLAB Editor window and edit the values for the 8 parameters in lines 23 to 30. The parameters are the same as those in the GUI version. Run "Hybtracking_script.m" and select the image and the output path directory. Choose the initial particle positions as shown in Supplementary Fig. 2.

3. Instructions for using the compiled version of HybTrack

Download "HybTrack_compiled_window.zip" or "HybTrack_compiled_mac.zip" from http://github.com/bhlee1117/HybTrack/. The compiled version of HybTrack does not require full

MATLAB. To run the compiled version, the user only needs to install MATLAB Runtime available at https://www.mathworks.com/products/compiler/matlab-runtime.html. The tracking process is the same as the GUI version of HybTrack.

Supplementary Figures

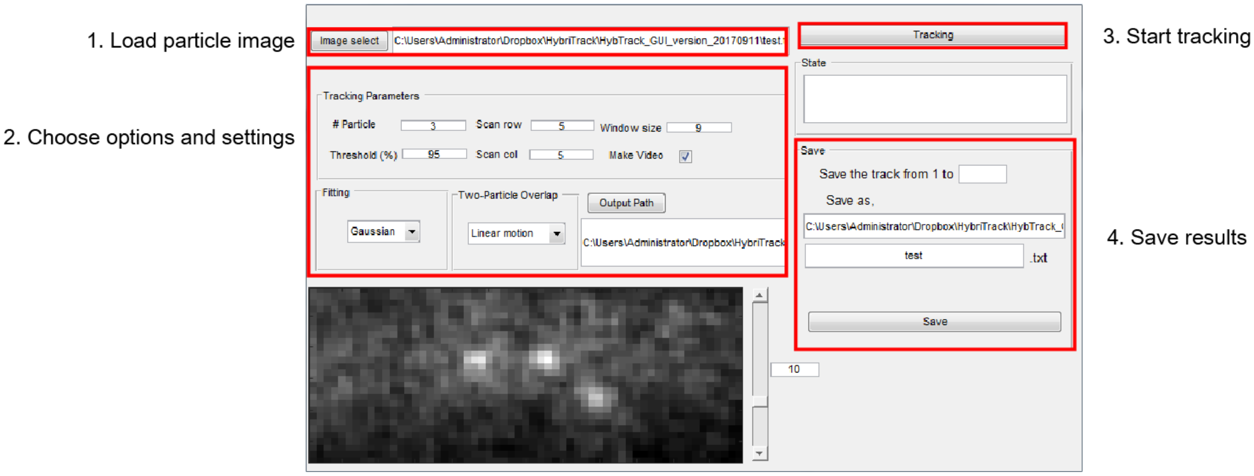

Supplementary Figure S1. Work flow of HybTrack. GUI interface of HybTrack is shown with brief instructions for using the software. The detailed instruction can be found in the Supplementary Note.

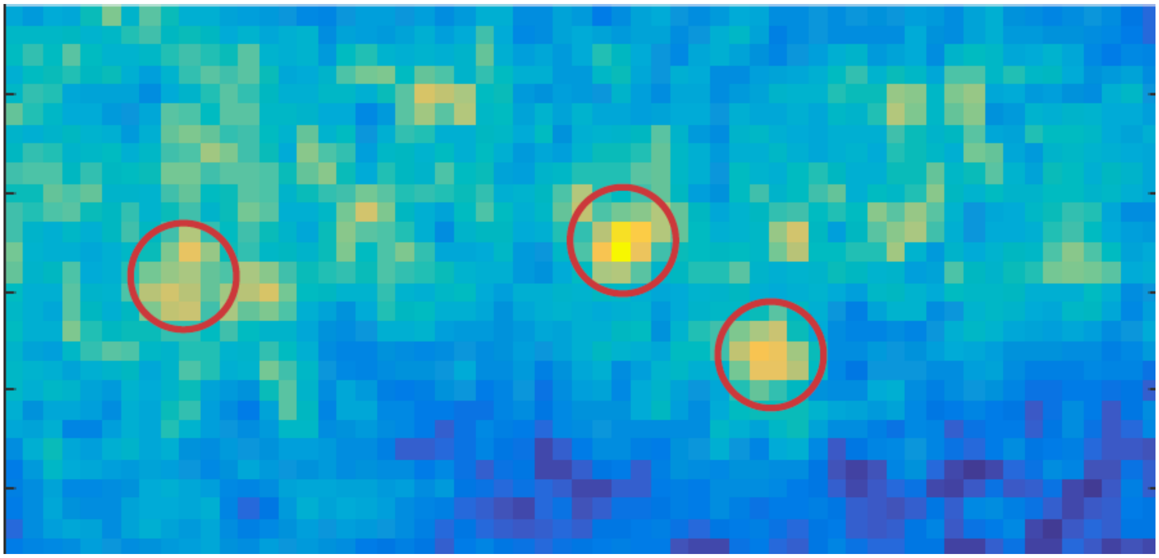

Supplementary Figure S2. Pop-up window for selecting the initial particle positions. For example, three positions (red circles) can be selected in "test.tif" image.

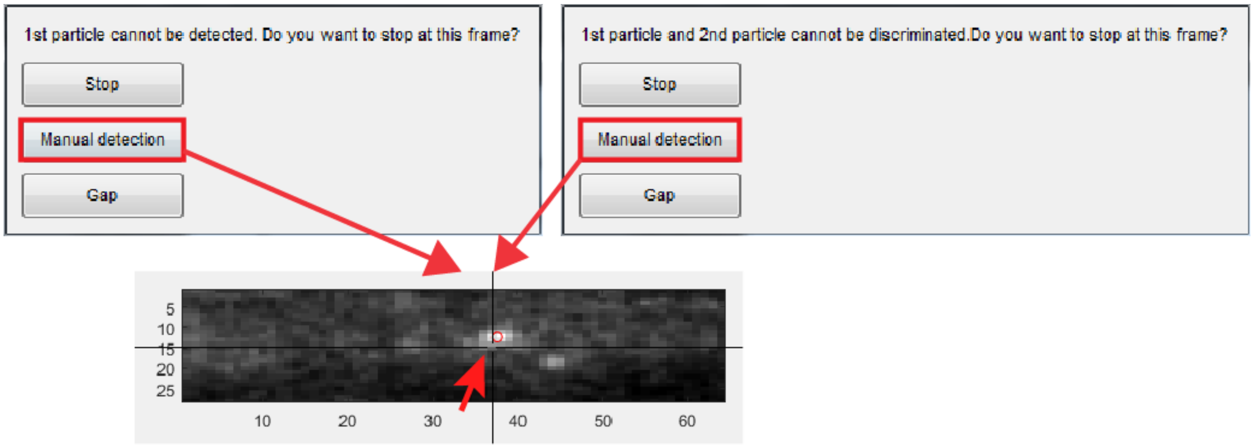

Supplementary Figure S3. Pop-up windows for manual tracking. There are two cases for which a manual tracking window pops up. The first one is when a local maximum is not found within the scan region using the given ***Threshold*** setting (upper left panel). The second case is when two particles are detected within the scan region (upper right panel). If the ***Manual detection*** option is selected, another pop-up window (lower panel) appears so that the user can select the position of the particles.

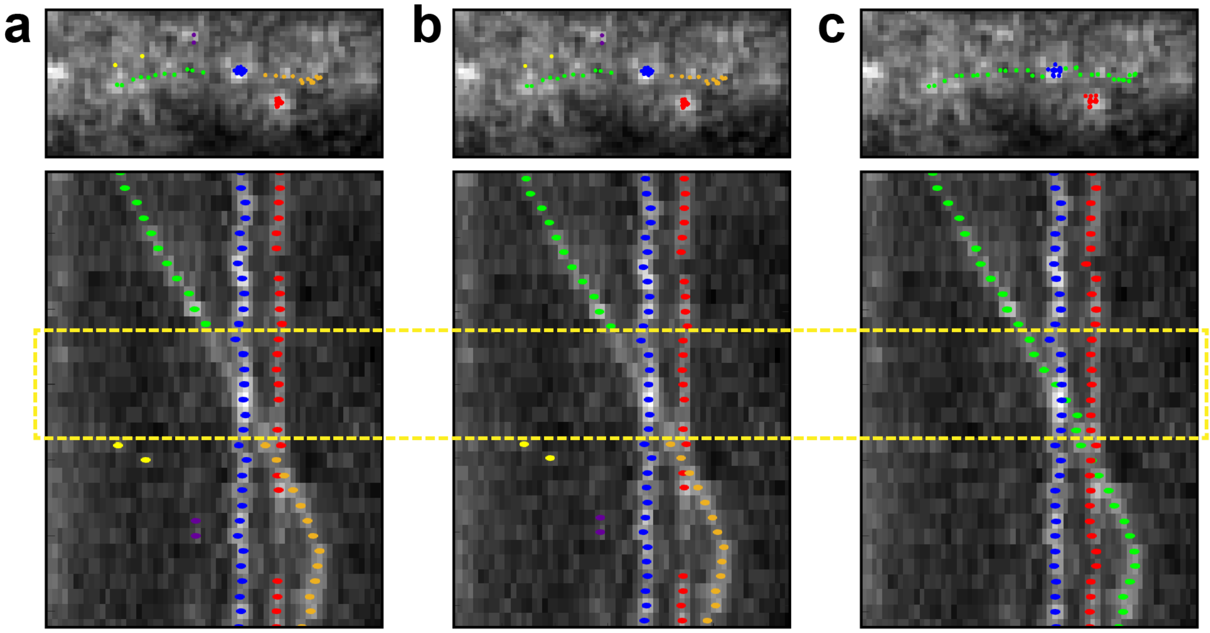

Supplementary Figure S4. Tracking particles with an overlapping event. **(a-c)** Tracking results from u-Track (a), TrackNTrace (b) and HybTrack (c). Particle trajectories obtained from each program are overlaid on the image (upper panels) and the kymograph (lower panels). Each trajectory is shown in a different color. In the image, two particles (green and blue) are overlapped during the time denoted by the yellow dotted box. Using the "linear motion" option in HybTrack, the two particles are successfully tracked without any manual detection procedure.

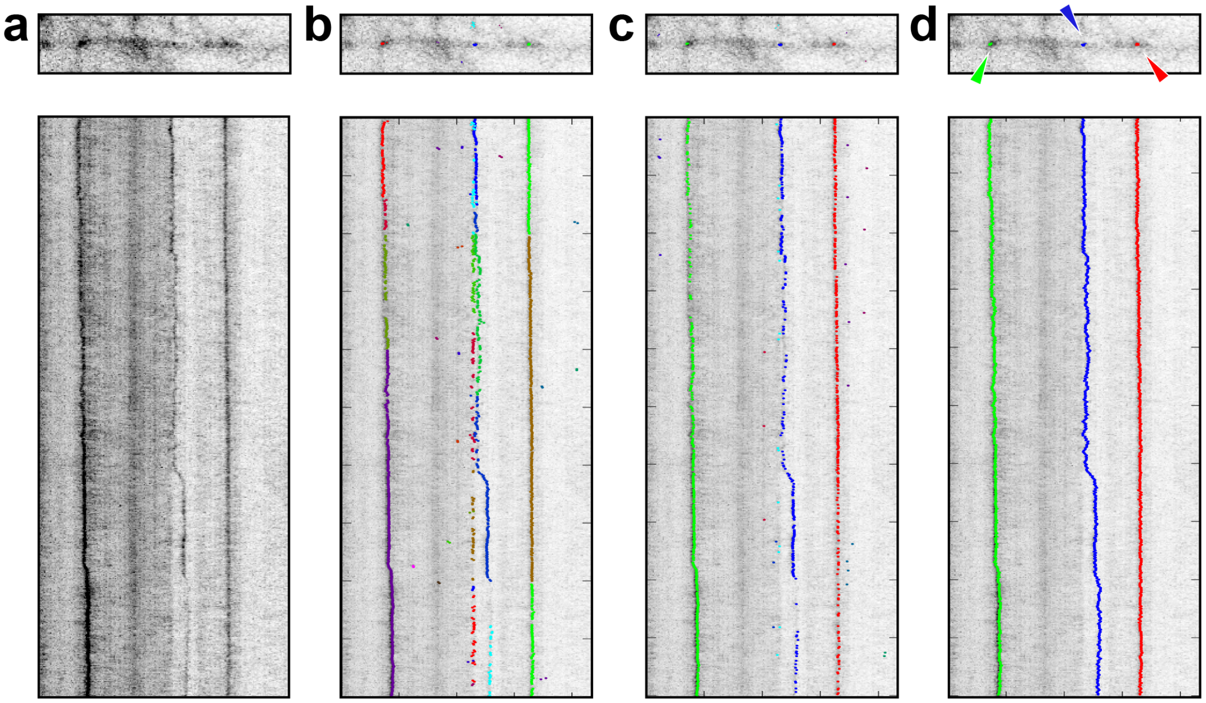

Supplementary Figure S5. Tracking particles with a low signal-to-noise ratio. (a) Image of mRNAs in a dendrite and kymograph (x-t) generated from the time-lapse image. **(b-d)** Tracking results from u-Track (b), TrackNTrace (c) and HybTrack (d). Particle trajectories obtained from each program are overlaid on the image (upper panels) and the kymograph (lower panels). Each trajectory is shown in a different color.

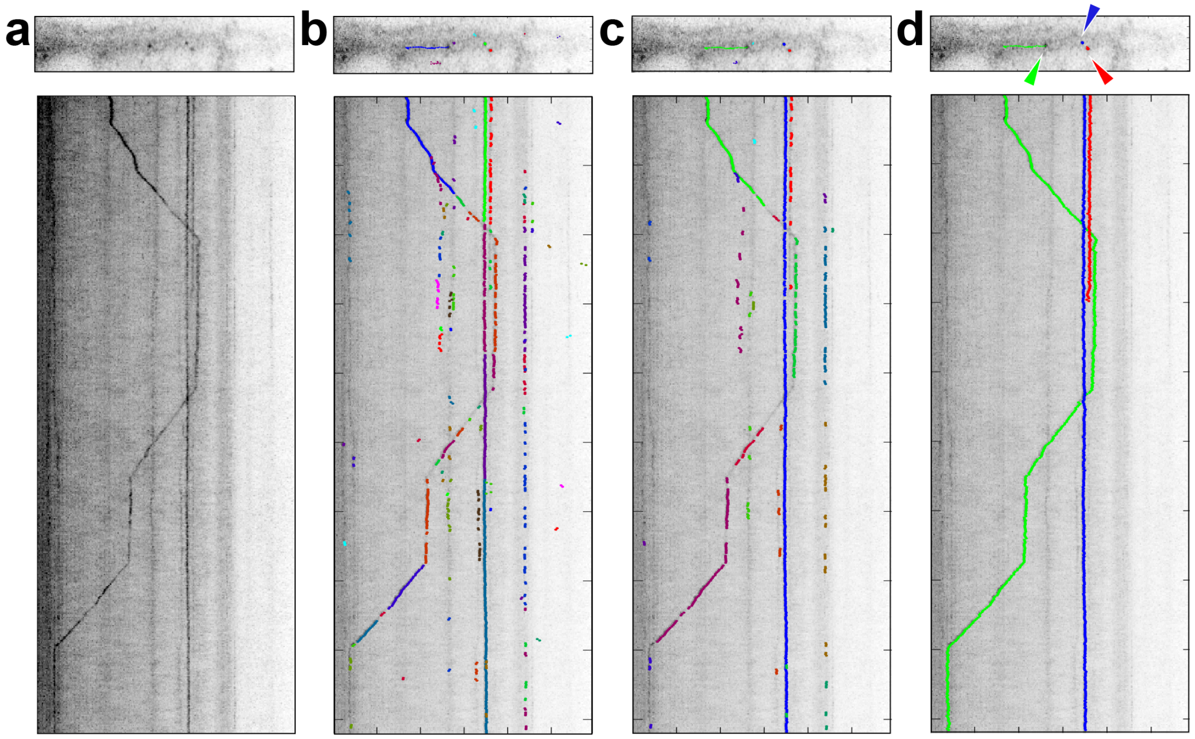

Supplementary Figure S6. Tracking the directed motion of the mRNAs. (a) Image of mRNAs in a dendrite and kymograph (x-t) generated from the time-lapse image. **(b-d)** Tracking results from u-Track (b), TrackNTrace (c) and HybTrack (d). Particle trajectories obtained from each program are overlaid on the image (upper panels) and the kymograph (lower panels). Each trajectory is shown in a different color. The full trajectories of three particles are successfully traced by using HybTrack with only 6 clicks of manual selection in the 460-frame-long time-lapse image.

Captions for Supplementary Movies

Supplementary Movie S1. Tracking movie of β-actin mRNAs in the dendrite shown in Fig. 2. Time-lapse images were acquired at 1 fps for 470 s. The Movie is played at 31 times real time speed. The upper panel shows detected mRNAs in the time-lapse image data. The middle panel shows the traces of mRNAs overlaid on the time-averaged image. The lower panel shows the traces of detected mRNAs on the kymograph.

Supplementary Movie S2. Tracking movie of β-actin mRNAs in the dendrite shown in Supplementary Fig. 4. This movie shows an overlapping event of mRNAs. Time-lapse images were acquired at 20 fps. The Movie is played at 1.5 times real time speed. The upper panel shows detected mRNAs in the time-lapse image data. The lower panel shows the traces of detected mRNAs overlaid on the time-averaged image.

Supplementary Movie S3. Tracking movie of β-actin mRNA in the dendrite shown in Supplementary Fig. 5. Time-lapse images were acquired at 20 fps for 75 s. The Movie is played at 1.5 times real time speed. The upper panel shows detected mRNAs in the time-lapse image data. The middle panel shows the traces of mRNAs overlaid on the time-averaged image. The lower panel shows the traces of detected mRNAs on the kymograph.

Supplementary Movie S4. Tracking movie of β-actin mRNA in the dendrite shown in Supplementary Fig. 6. Time-lapse images were acquired at 20 fps for 23 s. The Movie is played at 1.5 times real time speed. The upper panel shows detected mRNAs in the time-lapse image data. The middle panel shows the traces of mRNAs overlaid on the time-averaged image. The lower panel shows the traces of detected mRNAs on the kymograph.

Reference

Anthony, S. M. and S. Granick (2009). "Image Analysis with Rapid and Accurate Two-Dimensional Gaussian Fitting." Langmuir 25(14): 8152-8160.
